# Risk Factors Associated with Mortality in COVID-19 Hospitalized Patients: Data from the Middle East

**DOI:** 10.1155/2022/9617319

**Published:** 2022-08-16

**Authors:** Reema A. Karasneh, Basheer Y. Khassawneh, Sayer Al-Azzam, Abdel-Hameed Al-Mistarehi, William J. Lattyak, Motasem Aldiab, Suad Kabbaha, Syed Shahzad Hasan, Barbara R. Conway, Mamoon A. Aldeyab

**Affiliations:** ^1^Department of Basic Medical Sciences, Faculty of Medicine, Yarmouk University, Irbid, Jordan; ^2^Department of Internal Medicine, Faculty of Medicine, Jordan University of Science and Technology, Irbid, Jordan; ^3^Department of Clinical Pharmacy, Faculty of Pharmacy, Jordan University of Science and Technology, Irbid, Jordan; ^4^Department of Public Health and Family Medicine, Faculty of Medicine, Jordan University of Science and Technology, Irbid, Jordan; ^5^Scientific Computing Associates Corp., River Forest, IL, USA; ^6^Department of Computing, British Columbia Institute of Technology, Vancouver, Canada; ^7^Department of Health Research Methods, Evidence & Impact (HEI), McMaster University, Hamilton, Canada; ^8^Department of Pharmacy, School of Applied Sciences, University of Huddersfield, Huddersfield, UK; ^9^Institute of Skin Integrity and Infection Prevention, University of Huddersfield, Huddersfield, UK

## Abstract

This study aimed to assess the risk factors for COVID-19 mortality among hospitalized patients in Jordan. All COVID-19 patients admitted to a tertiary hospital in Jordan from September 20, 2020, to August 8, 2021, were included in this study. Demographics, clinical characteristics, comorbidities, and laboratory results were extracted from the patients' electronic records. Multivariable logistic and machine learning (ML) methods were used to study variable importance. Out of 1,613 COVID-19 patients, 1,004 (62.2%) were discharged from the hospital (survived), while 609 (37.8%) died. Patients who were of elderly age (>65 years) (OR, 2.01; 95% CI, 1.28–3.16), current smokers (OR, 1.61; 95%CI, 1.17–2.23), and had severe or critical illness at admission ((OR, 1.56; 95%CI, 1.05–2.32) (OR, 2.94; 95%CI, 2.02–4.27); respectively), were at higher risk of mortality. Comorbidities including chronic kidney disease (OR, 2.90; 95% CI, 1.90–4.43), deep venous thrombosis (OR, 2.62; 95% CI, 1.08–6.35), malignancy (OR, 2.22; 95% CI, 1.46–3.38), diabetes (OR, 1.31; 95% CI, 1.04–1.65), and heart failure (OR, 1.51; 95% CI, 1.02–2.23) were significantly associated with increased risk of mortality. Laboratory abnormalities associated with mortality included hypernatremia (OR, 11.37; 95% CI, 4.33–29.81), elevated aspartate aminotransferase (OR, 1.81; 95% CI, 1.42–2.31), hypoalbuminemia (OR, 1.75; 95% CI, 1.37–2.25), and low platelets level (OR, 1.43; 95% CI, 1.05–1.95). Several demographic, clinical, and laboratory risk factors for COVID-19 mortality were identified. This study is the first to examine the risk factors associated with mortality using ML methods in the Middle East. This will contribute to a better understanding of the impact of the disease and improve the outcome of the pandemic worldwide.

## 1. Introduction

Severe acute respiratory syndrome coronavirus 2 (SARS-CoV-2), or as it was provisionally named, the 2019 novel coronavirus (2019-nCoV) disease (COVID-19), is a highly contagious viral illness [1]. It has imposed catastrophic global morbidity and mortality burdens with more than 340 million cases and 5 million deaths [2, 3]. In particular, excess mortality of over one million was observed in more than 100 countries in 2020 [4]. Furthermore, a recent study has estimated an excess of 375,235 deaths from direct or indirect effects of COVID-19 [5]. Therefore, even survivors of COVID-19 may develop long-term effects and clinical consequences such as heart and lung damage leading to possible delayed death [6].

The evolution of this pandemic has required the urgent expansion of public health efforts to understand its epidemiology better and identify its impact [7]. Identifying COVID-19 impact requires the elucidation of the spectrum of its clinical severity, including the recognition of potential risk factors for severe illness or death [7]. Consequently, since its emergence, several epidemiological studies have been conducted and shown a significant association between COVID-19 severity and death with demographic characteristics, preexisting comorbidities, and other factors [8, 9].

Several demographic risk factors were observed for COVID-19 severity and mortality including older age and male gender [8, 9]. Ethnicity was also found to be associated with COVID-19 mortality as observed in a study that used OpenSAFELY in which Black and South Asian patients had increased risk of mortality when compared with patients of White ethnicity (adjusted hazard ratio [aHR] 1.48; 95% CI, 1.29–1.69; and 1.45; 95% CI, 1.32–1.58, respectively) [10]. In addition, educational attainment was significantly associated with a lower risk of COVID-19 severity (odds ratio [OR] per one standard deviation increase in years of schooling, 0.540; 95% CI, 0.376–0.777, *P*=0.0009) in a European study that used Mendelian randomization (MR) approach [11].

Besides, higher scores of the Charlson Comorbidity Index (CCI) were positively associated with mortality in COVID-19 patients with each point increase in the CCI score increasing the risk of death by 2.5% [12]. In fact, comorbidities in COVID-19 patients were found to hasten the progression of the infection that usually ends with patient death [13]. These findings suggest targeting this group of patients in vaccination plans and enhancing earlier identification and treatment to obtain better outcomes [12, 13]. In addition, a multicenter study has used laboratory biomarkers for the establishment of cutoff points and building a risk score ranging from 0 to 30 points. The study found that moderate (12–18 points) and high-risk patients (≥19 points) were positively associated with COVID-19 mortality (OR 4.75; CI 95%, 2.60–8.68 and 23.86; CI 95%, 13.61–41.84, respectively) [14].

These studies have provided a better understanding of COVID-19 impact and have raised several questions that require further investigation. For instance, a recent population-based study has found that heart failure and ischemic heart disease increased the risk of mortality, while atrial fibrillation and hypertension did not, suggesting potential protective effects of comedications [15]. In addition, other studies have investigated COVID-19 impact on patients with particular diseases or conditions in addition to healthy patients [16–20].

Most studies have been conducted in China, Italy, the USA, and other developed high-income countries, while studies from low- and middle-income developing countries in the Middle East are scarce [21, 22]. Therefore, the complete impact of COVID-19 is not thoroughly evaluated, and further studies are required to fill the gap [22]. In this study, we aimed to describe the characteristics of hospitalized patients with COVID-19 and identify the risk factors associated with in-hospital mortality for patients with COVID-19 in Jordan.

## 2. Materials and Methods

### 2.1. Data Source and Study Design

This study was conducted using data from King Abdullah University Hospital (KAUH) in Jordan, which has a 750-bed capacity that can be increased to 900 in emergency situations. It is a tertiary hospital and is considered the largest medical facility in the north of the country. All positive COVID-19 patients in the north of Jordan were referred to this hospital, including asymptomatic and mild cases at the beginning of the pandemic. However, hospitalization criteria have changed since August 2020, with only moderate, severe, or critical cases being hospitalized.

A cohort of COVID-19 patients was identified from inpatients' electronic records based on polymerase chain reaction (PCR) positive test results for each referred patient at the hospital. The study period was between September 20, 2020, and August 8, 2021. Cohort members younger than 18 years and those with asymptomatic infection or mild illness were excluded.

### 2.2. Study Variables

The patients' organized clinical data, including vital signs, radiological findings, comorbidities, and hospitalization course and outcomes were identified from electronic hospital records. Data also included age, gender, smoking status, height, weight, and laboratory results. Body mass index (BMI) was calculated based on the equation of BMI = weight(kg)/height^2^(m^2^) and categorized based on the World Health Organization (WHO) classification [23]. Patients with BMI below 18.0 kg/m^2^ were considered underweight, 18.5–24.9 kg/m^2^ were of normal weight, 25.0–29.9 kg/m^2^ were overweight, and patients with BMI equal to or more than 30.0 kg/m^2^ were considered obese. Comorbidities were identified based on related International Classification of Diseases (ICD) codes, and laboratory results were interpreted based on the reference values of the hospital laboratory. The patient severity status at admission was classified according to the National Institute of Health (NIH) Clinical Spectrum of SARS-CoV-2 Infection [24]. Patients with a positive test and no symptoms were considered “asymptomatic” while if any of the various signs and symptoms of COVID-19 were observed with no shortness of breath, dyspnea, or abnormal chest imaging then these were considered to have “mild illness.” “Moderate illness” individuals included those who showed evidence of lower respiratory disease during clinical assessment or imaging and who had an oxygen saturation (SpO2) ≥94% on room air at sea level. Patients who had SpO2 <94%, a ratio of arterial partial pressure of oxygen to fraction of inspired oxygen (PaO2/FiO2) <300 mm Hg, a respiratory rate >30 breaths/min, or lung infiltrates >50% were considered of “severe illness.” “Critically ill” patients included those who had respiratory failure, septic shock, and/or multiple organ dysfunction.

### 2.3. Statistical Analysis

Analysis began with a distributional analysis of patients' characteristics. All categorical data were encoded using a one-hot approach which transforms categorical variables into binary vectors. Summary tables were created to examine the proportion of inpatients with COVID-19 by age group, gender, and clinical characteristics. The mortality rates associated with each characteristic were analyzed using *χ*2 tests to evaluate statistical differences in the frequencies of the categorical groups. In addition to ratio testing, we used univariate logistic regression to produce the odds ratio and statistical significance for each one-hot transformed characteristic. A 2-sided *P* ≤ 0.05 was considered statistically significant.

After examining the variable importance of each characteristic with the increased mortality rate for COVID-19 inpatients, multivariable logistics and machine learning (ML) methods were used to study the variable importance in a comprehensive manner concerning classification modeling. Variable importance is generally defined as the sum of the decrease in error when the variable is included in the model. For linear models, variable importance is based on the absolute t-value of each model parameter used. MARS models look at reductions in the modified generalized cross-validation (MGCV) estimate of error.

(1) $$\begin{array}{c} \text{MGCV}=\frac{\left[\left(1/N\right)\sum_{i=1}^{N}{\left\{y_{i}-\hat{f}\left(X\right)\right.}^{2}\right]}{\left[1-{\left[C{\left(M\right)}^{*}/N\right]}^{2}\right]}, \end{array}$$

where N is the number of observations, $\hat{f}\left(X_{i}\right)\equiv {\hat{y}}_{i}$ and *C*(*M*)^*∗*^ is a complexity penalty.

The relative importance is the variable importance divided by the highest variable importance value so that values are scaled between 0 and 1. This makes it convenient to compare and combine variable performance across multiple methods.

The ML methods considered were Random Forest (RF), Multivariate Adaptive Regression Splines (MARS), K-Nearest Neighbor (KNN), Extreme Gradient Boosting (XGB), and Classification and Regression Trees (CART). There are other ML methods and classification methods that can be entertained in addition to the methods employed here. For example, a stepwise regression method can be used to directly eliminate model parameters in the model given an overparameterized starting model. However, we feel the methods selected are among the most consistent in producing classification accuracy.

In terms of the MARS model notation, this is written *y*=*α*′*c*_1_(*x* − *τ*^*∗*^) ± *c*_2_(*τ*^*∗*^ − *x*)_+_+*e*, where *τ*^*∗*^ is the threshold value for the *x* predictor variable, and (·)+ are the spline functions which take on the value 0 if the expression inside (·)+ is negative or its actual value if the expression inside (·)+ is >0. The other considered ML methods are based on classification trees differing by an algorithmic implementation that recursively split variables. These algorithms are quite involved and would take considerable space to describe here and recommend further information to be found in the work Generalized Additive Models by Hastie and Tibshirani [25].

A ten-fold cross-validation method was used to train the ML methods and extract the combined relative importance of the variables identified in each fold. The results from the ten folds were then combined to produce a collective measure of variable importance by the number of times the variable appeared across folds and the magnitude of the variable predictive power. To combine variable importance across multiple methods, the importance measures were scaled between 100 and 0. The variables' importance for all methods were then combined into a single importance measure and were presented using a lollipop chart. The reduced set of variables was then used to refine a multivariable logistic regression model that can quantify potential risk factors that result in death, adjusted for other confounding characteristics. The backward stepwise variable selection method was used to determine the variables included in the final multivariable logistic regression model. A significant level of *P* < 0.05 was used to retain variables in the final logistic model. To assess the classification accuracy of the final multivariable logistic model compared to the other ML methods, the dataset was randomly partitioned into a training set (70% of data) and a testing set (30% of the data) using a stratified method so that the distributions are as consistent as possible. A panel of receiver operator characteristic (ROC) charts was created. The ROC charts show the performance of a classification model at all classification thresholds using the holdout testing set. The curve plots the True Positive Classification Rate versus the False Positive Classification Rate. The area under the curve (AUC) is an aggregate measure of performance across all possible classification thresholds. All analyses were carried out using the 4.1.0 release of the *R* package.

## 3. Results

Over the study period, a total of 1,613 patients with confirmed COVID-19 who were admitted to KAUH met the inclusion criteria and were included in this study. Of these patients, 1,004 (62.2%) were discharged from the hospital (survived), and 609 (37.8%) died (deceased).  Table 1 shows the characteristics, comorbidities, and disease severity of the patients by their hospitalization outcome (survived or deceased). Among all patients, 945 (58.6%) were male, 669 (41.5%) were obese (BMI ≥30 kg/m^2^), and 963 (59.7%) and 802 (49.7%) had hypertension and diabetes, respectively. Surviving patients were of a younger age group (18–40 years old), nonsmokers, and with moderate severity on admission (patients with lower respiratory disease and who have SpO2 ≥94%). Higher proportions of deceased patients compared to the survived patients were observed among those with heart failure (*n* = 73, 51.0%; *p* value = 0.001), cerebrovascular accident (CVA) (*n* = 61, 55.5%; *p* value <0.001), chronic kidney disease (CKD) (*n* = 85, 66.9%; *p* value <0.001), immunocompromised (*n* = 37, 56.9%; *p* value = 0.002), and malignancy (*n* = 65, 54.6%; *p* value <0.001).

The laboratory findings from COVID-19 patients are shown in Table 2. Among all patients, a large proportion had high levels of C-reactive protein (CRP) (*n* = 1,129, 70.0%), D-dimer (*n* = 1, 192, 73.9%), and lactate dehydrogenase (LDH) (*n* = 1,112, 68.9%). Higher proportions of deceased patients compared to survived patients had hypernatremia (*n* = 43, 89.6%; *p* value <0.001), hyperkalemia (*n* = 61, 54.5%; *p* value <0.001), and high troponin level (*n* = 15, 62.5%; *p* value = 0.041).

Multivariate logistic regression showed that elderly age (>65 years old) (OR, 2.01; 95% CI, 1.28–3.16; *p* value = 0.003) and current smoking status (OR, 1.61; 95% CI, 1.17–2.23; *p* value = 0.004) were strongly associated with in-hospital mortality (Table 3). Odds of severity status were almost doubled for the risk of mortality between severe (OR, 1.56; 95% CI, 1.05–2.32; *p* value <0.001) and critical (OR, 2.94; 95% CI, 2.02–4.27; *p* value <0.001) cases of COVID-19 patients on admission. Although significant, higher odds for in-hospital mortality were observed for CKD (OR, 2.90; 95% CI, 1.90–4.43; *p* value <0.001), DVT (OR, 2.62; 95%CI, 1.08–6.35; *p* value = 0.033), and malignancy (OR, 2.22; 95% CI, 1.46–3.38; *p* value <0.001) compared to diabetes (OR, 1.31; 95% CI, 1.04–1.65; *p* value = 0.022) and heart failure (OR, 1.51; 95% CI, 1.02–2.23; *p* value = 0.041). Hypernatremia level was the risk factor most strongly associated with mortality among COVID-19 patients (OR, 11.37; 95% CI, 4.33–29.81; *p* value <0.001). Other significantly associated lab results with mortality included high AST (OR, 1.81; 95% CI, 1.42–2.31; *p* value <0.001), hypoalbuminemia levels (OR, 1.75; 95% CI, 1.37–2.25; *p* value <0.001), and low platelets level (OR, 1.43; 95% CI, 1.05–1.95; *p* value = 0.024).

**Figure 1:**
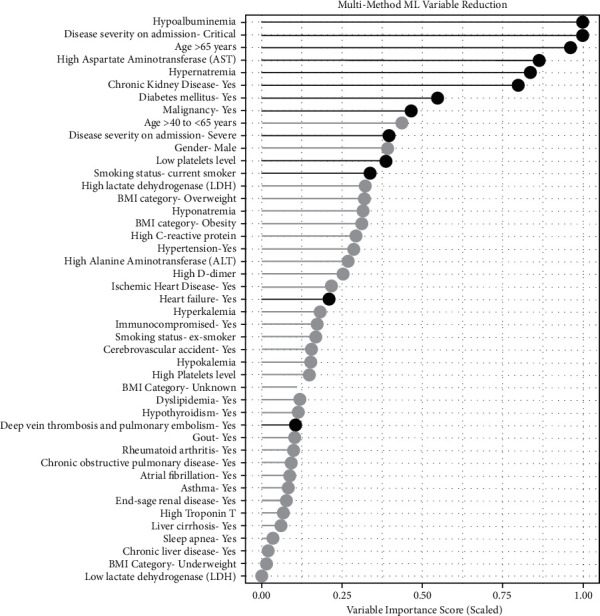
Variable reduction based on the consensus variable importance extracted from multiple ML methods. Variables highlighted in black color represent variables selected for the final multiple logistic regression model.

Figure 1 shows the variable reduction based on the consensus variable importance extracted from multiple ML methods. Logistic regression was then applied to the consensus. The variables selected for the final multiple logistic regression model are highlighted in black color.

**Figure 2:**
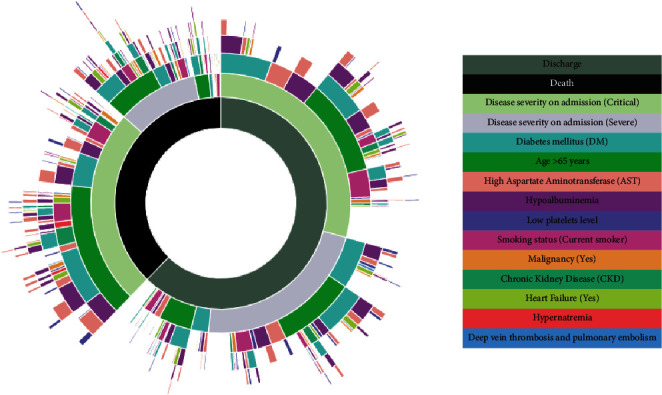
Sunburst chart with nested rings illustrating the hierarchical breakdown of identified risk factors segmented by patients' outcome, that is, death versus discharge.

A sunburst chart with nested rings illustrating the hierarchical breakdown of identified risk factors segmented by patients' outcome, that is, death versus discharge, is shown in Figure 2.

**Figure 3:**
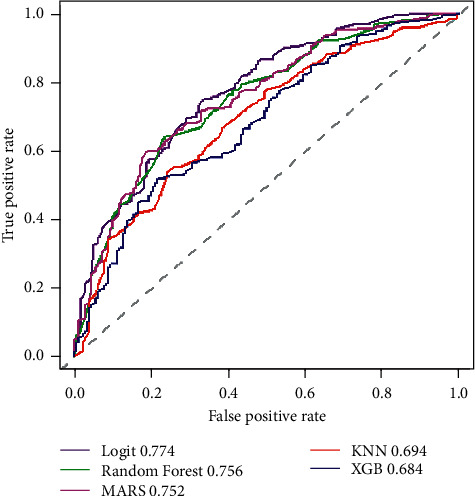
Receiver Operator Characteristic (ROC) charts for the Multiple Logistic Regression model compared to the multiple ML methods used for variable reduction.

The panel of Receiver Operator Characteristic (ROC) chart is shown in Figure 3 and ordered by predictive accuracy. The multivariable logistic regression model has the best predictive classification power among the other methods considered.

## 4. Discussion

In this study, we observed a high hospital mortality (37.8%) among hospitalized COVID-19 patients. Almost half of the deceased patients (45.4%) had critical disease on admission. The study identified several risk factors for increased hospital mortality including older age, current smoking status, critical disease on admission, the presence of comorbidities, and initial laboratory derangements. To our knowledge, this study is the first to examine the risk factors associated with mortality in the Middle East.

Several studies have found that older age was significantly associated with increased mortality of COVID-19 [17, 26–29]. Consistent with our findings, a meta-analysis of 42 studies comprising 423,117 patients showed an increased risk of mortality among older people (pooled OR 2.61; 95% CI 1.75–3.47) [26]. Specifically, among 16 countries, COVID-19 patients aged 65 or older had a 62-fold higher risk of mortality than younger age groups (IRR = 62.1; 95% CI = 59.7, 64.7) [30]. The increase in mortality among the elderly may be attributed to the high prevalence of comorbidities [9]. However, a study that used UK Biobank data and included 470,034 participants concluded that, although to a lower extent, healthy elderly COVID-19 patients still have an independently increased risk of mortality [31]. This would suggest a role of other age-related factors in COVID-19 mortality, such as a decreased reserve capacity of vital organs or weaker immune defenses [32].

Our results were not consistent with the findings of other studies that showed evidence of increased mortality in male COVID-19 patients [33–36]. However, most of these studies were at the early stages of COVID-19 and were based on records that under urgent circumstances may have had missing data and used unadjusted risk estimates [33]. On the other hand, female cases and deaths from COVID-19 may have been underreported due to social norms of lower access to healthcare [37]. Therefore, gender differences should be further investigated to avoid bias in COVID-19 treatment, particularly with the similar risk of dying observed between males and females in severe cases [38, 39].

A recent systematic review and meta-analysis of 186 studies representing 210,447 deaths among 1,304,587 patients with COVID-19, found a significant increase in mortality among patients with diabetes (summary relative risk (SRR) = 1.54; 95% CI 1.44–1.64), hypertension (SRR = 1.42; 95% CI 1.30–1.54), obesity (SSR = 1.45; 95% CI 1.31–1.61), and smoking (SRR = 1.28; 95% CI 1.17 to 1.40 forever smoking, SRR = 1.29; 95% CI 1.03 to 1.62 for current smoking, and SRR = 1.25; 95% CI 1.11 to 1.42 for ex-smokers compared with nonsmokers) [40]. However, in our study, obesity and hypertension did not appear statistically significant in the final model, and an ex-smoker status was no longer significant when conducting the multivariate analyses. Similar to our findings, CKD was significantly associated with an increased risk of death (pooled OR = 5.58; 95% CI 3.27–9.54) [41]. Other comorbidities, including HF, history of DVT and PE, and malignancy were also found to be significantly associated with increased mortality in COVID-19 patients in several studies [42–46].

In health outcome predictive evaluation (HOPE) for COVID-19 registry analysis, both hypernatremia (OR 2.38, 95% CI 1.18–4.78; *p*=0.015) and hyponatremia (OR 1.5, 95% CI 1.08–2.09; *p*=0.016) were found to be independently associated with COVID-19 mortality [47]. This is consistent with our findings for hypernatremia but not hyponatremia. However, the admittance frequency with hyponatremia (20.5%) was higher than that with hypernatremia (3.7%), as observed in our study [47]. Besides being consistent with our findings, several studies have identified hypoalbuminemia as a predictor of mortality among admitted COVID-19 patients, suggesting the determination of serum albumin on admission may be useful and there could be a potential therapeutic value for albumin infusion in COVID‐19 management [48–50]. Similarly, low platelet level was found to be significantly associated with COVID-19 mortality, as observed in our study [51, 52]. However, the prognostic value of the thrombocytopenia therapeutic approach appears to be complicated and careful consideration is advised [53].

This study was relatively large and included data from the Middle East with PCR-confirmed COVID-19 cases. It used in-patient record data from a tertiary hospital to reflect the reality of COVID-19 clinical management in the region. However, the observational nature of this study does not have a temporal sequence and therefore cannot lead to causal associations.

## 5. Conclusions

Several demographic, clinical, and laboratory risk factors for COVID-19 mortality were identified, including severity status on admission. Further studies in real-life settings are required, particularly in the region, to identify early predictors and provide better management of the illness to control the pandemic and reduce its impact.

## Figures and Tables

**Table 1:** Characteristics, comorbidities, and disease severity in patients with COVID-19 (*N*  = 1,613).

Characteristics	Overall sample (*N* = 1,613)	Survived (*N* = 1,004)	Deceased (*N* = 609)	*p* value
	No. (%)	No. (%)	No. (%)	
Age, years				
18–40	148 (9.2)	115 (77.7)	33 (22.3)	<0.001
41–65	737 (45.7)	520 (70.6)	217 (29.4)	
>65	728 (45.1)	369 (50.7)	359 (49.3)	

Gender				
Female	668 (41.4)	427 (63.9)	241 (36.1)	0.264
Male	945 (58.6)	577 (61.1)	368 (38.9)	

Body mass index, (kg/m^2^)				
Underweight (<18.5)	4 (0.2)	2 (50.0)	2 (50.0)	0.221
Normal (18.5–25)	227 (14.1)	134 (59.0)	93 (41.0)	
Overweight (25–30)	554 (34.3)	357 (64.4)	197 (35.6)	
Obese (>30)	669 (41.5)	403 (60.2)	266 (39.8)	
Unknown	159 (9.9)	108 (67.9)	51 (32.1)	

Smoking status				
Current smoker	223 (13.8)	123 (55.2)	100 (44.8)	0.009
Ex-smoker	191 (11.8)	107 (56.0)	84 (44.0)	
Nonsmoker	1,199 (74.3)	774 (64.6)	425 (35.4)	

Comorbidities				
Hypertension	963 (59.7)	558 (57.9)	405 (42.1)	<0.001
Diabetes mellitus	802 (49.7)	467 (58.2)	335 (41.8)	0.001
Dyslipidemia	86 (5.3)	52 (60.5)	34 (39.5)	0.814
Ischemic heart disease	291 (18.0)	156 (53.6)	135 (46.4)	0.001
Atrial fibrillation	60 (3.7)	35 (58.3)	25 (41.7)	0.616
Heart failure	143 (8.9)	70 (49.0)	73 (51.0)	0.001
Asthma	49 (3.0)	31 (63.3)	18 (36.7)	0.999
Chronic obstructive pulmonary disease	17 (1.1)	7 (41.2)	10 (58.8)	0.121
Sleep apnea	12 (0.7)	7 (58.3)	5 (41.7)	0.999
Cerebrovascular accident (old stroke)	110 (6.8)	49 (44.5)	61 (55.5)	<0.001
Chronic kidney disease	127 (7.9)	42 (33.1)	85 (66.9)	<0.001
End-stage renal disease	32 (2.0)	18 (56.3)	14 (43.8)	0.576
Chronic liver disease	4 (0.2)	1 (25.0)	3 (75.0)	0.307
Liver cirrhosis	2 (0.1)	0 (0.0)	2 (100.0)	0.277
Rheumatoid arthritis	21 (1.3)	11 (52.4)	10 (47.6)	0.476
History of venous thromboembolism	26 (1.6)	11 (42.3)	15 (57.7)	0.056
Immunocompromised	65 (4.0)	28 (43.1)	37 (56.9)	0.002
Malignancy	119 (7.4)	54 (45.4)	65 (54.6)	<0.001
Hypothyroidism	78 (4.8)	43 (55.1)	35 (44.9)	0.227
Gout	74 (4.6)	46 (62.2)	28 (37.8)	0.999

Severity on admission				
Moderate	226 (14.0)	173 (76.5)	53 (23.5)	<0.001
Severe	522 (32.4)	359 (68.8)	163 (31.2)	
Critical	865 (53.6)	472 (54.6)	393 (45.4)	

**Table 2:** Laboratory characteristics of COVID-19 patients (*N*  = 1,613).

Laboratory test	Overall sample (*n* = 1,6013)	Survived (*N* = 1,004)	Deceased (*N* = 609)	*p* value
	No. (%)	No. (%)	No. (%)	
C-reactive protein (CRP) ^*∗*^				
High	1,129 (70.0)	685 (60.7)	444 (39.3)	0.004
Low	28 (1.7)	25 (89.3)	3 (10.7)	
Unknown	456 (28.3)	294 (64.5)	162 (35.5)	

D-dimer ^*∗*^				
High	1, 192 (73.9)	724 (60.7)	468 (39.3)	0.038
Low	11 (0.7)	5 (45.5)	6 (54.5)	
Unknown	410 (25.4)	275 (67.1)	135 (32.9)	

Lactate dehydrogenase (LDH) ^*∗*^				
High	1.112 (68.9)	679 (61.1)	433 (38.9)	0.071
Normal	33 (2.0)	27 (81.8)	6 (18.2)	
Low	1 (0.1)	1 (100.0)	0 (0.0)	
Unknown	467 (29.0)	297 (63.6)	170 (36.4)	

Sodium ^*∗*^				
Hypernatremia	48 (3.0)	5 (10.4)	43 (89.6)	<0.001
Normal	862 (53.4)	544 (63.1)	318 (36.9)	
Hyponatremia	470 (29.1)	285 (60.6)	185 (39.4)	
Unknown	233 (14.4)	170 (73.0)	63 (27.0)	

Potassium ^*∗*^				
Hyperkalemia	112 (6.9)	51 (45.5)	61 (54.5)	<0.001
Normal	1,134 (70.3)	709 (62.5)	425 (37.5)	
Hypokalemia	134 (8.3)	74 (55.2)	60 (44.8)	
Unknown	233 (14.4)	170 (73.0)	63 (27.0)	

Albumin ^*∗*^				
Normal	616 (38.2)	443 (71.9)	173 (28.1)	<0.001
Hypoalbuminemia	747 (46.3)	380 (50.9)	367 (49.1)	
Unknown	250 (15.5)	181 (72.4)	69 (27.6)	

Alanine transaminase (ALT) ^*∗*^				
High	290 (18.0)	171 (59.0)	119 (41.0)	<0.001
Normal	1,103 (68.4)	669 (60.7)	434 (39.3)	
Unknown	220 (13.6)	164 (74.5)	56 (25.5)	

Aspartate aminotransferase (AST) ^*∗*^				
High	589 (36.5)	307 (52.1)	282 (47.9)	<0.001
Normal	802 (49.7)	531 (66.2)	271 (33.8)	
Unknown	222 (13.8)	166 (74.8)	56 (25.2)	

Troponin ^*∗*^				
High	24 (1.5)	9 (37.5)	15 (62.5)	0.041
Normal	520 (32.2)	327 (62.9)	193 (37.1)	
Unknown	1,069 (66.3)	668 (62.5)	401 (37.5)	

Platelets ^*∗*^				
High	139 (8.6)	91 (65.5)	48 (34.5)	<0.001
Normal	1,094 (67.8)	684 (62.5)	410 (37.5)	
Low	254 (15.7)	133 (52.4)	121 (47.6)	
Unknown	126 (7.8)	96 (76.2)	30 (23.8)	

^*∗*^Normal levels: CRP: 0.0–5.0 mg/L; D-dimer: 0.1–0.5 ug/ml; LDH: 264.6–441.0 U/L; sodium: 135–145 mmol/L; potassium: 3.7–5.4 mmol/L; albumin: 35–52 g/L; ALT: 0.0–41.0 U/L; AST: 0.0–40.0 U/L; troponin: 0.000–0.20 ng/ml; platelets: 150 × 10^3^–400 × 10^3^/mm^3^.

**Table 3:** Multivariate logistic regression results assess the risk factors associated with in-hospital mortality among inpatients with confirmed COVID-19.

Covariate	Univariate regression		Multivariate regression	
	Or (95% CI)	*p* value	Or (95% CI)	*p* value
Age, years				
18–40 (reference)				
41–65	1.45 (0.96–2.21)	0.079	1.06 (0.68–1.67)	0.786
>65	3.39 (2.24–5.13)	<0.001	2.01 (1.28–3.16)	0.003

Severity				
Moderate (reference)				
Severe	1.48 (1.04–2.12)	0.032	1.56 (1.05–2.32)	<0.001
Critical	2.72 (1.94–3.80)	<0.001	2.94 (2.02–4.27)	<0.001

Smoking status				
Nonsmoker (reference)				
Current smoker	1.48 (1.11–1.98)	0.008	1.61 (1.17–2.23)	0.004
Ex-smoker	1.43 (1.05–1.95)	0.024	1.31 (0.94–1.84)	0.115

Comorbidities				
Chronic kidney disease, yes vs. no	3.72 (2.53–5.46)	<0.001	2.90 (1.90–4.43)	<0.001
Diabetes miletus, yes vs. no	1.41 (1.15–1.72)	0.001	1.31 (1.04–1.65)	0.022
Heart failure, yes vs. no	1.82 (1.29–2.57)	0.001	1.51 (1.02–2.23)	0.041
History of DVT/PE, yes vs. no	2.28 (1.04–5.00)	0.040	2.62 (1.08–6.35)	0.033
Malignancy, yes vs. no	2.10 (1.44–3.06)	<0.001	2.221 (1.46–3.38)	<0.001

Lab results				
Sodium normal (reference)				
Hypernatremia	14.71 (5.77–37.52)	<0.001	11.37 (4.33–29.81)	<0.001
Hyponatremia	1.11 (0.88–1.40)	0.374	0.91 (0.71–1.18)	0.474
Albumin normal (reference)				
Hypoalbuminemia	2.47 (1.97–3.10)	<0.001	1.75 (1.37–2.25)	<0.001
Normal AST level (reference)				
High AST level	1.80 (1.45–2.24)	<0.001	1.81 (1.42–2.31)	<0.001
Platelets normal (reference)				
Platelets high	0.88 (0.61–1.28)	0.499	0.84 (0.56–1.27)	0.402
Platelets low	1.52 (1.15–2.00)	0.003	1.43 (1.05–1.95)	0.024

## Data Availability

Data are available based upon request from KAUH.

## Additional Points

*Problem Statement.* What is already known about this topic? (i)COVID-19 is a highly contagious illness that causes high mortality worldwide. (ii) Mortality in COVID-19 patients has been associated with several risk factors including demographics and preexisting comorbidities. (iii) Similar studies using real data in the Middle East are scarce. What does this article add? (i) The study is the first to identify potential risk factors for COVID-19 mortality in the Middle East. (ii) Machine learning methods were used to study the variable's importance. (iii) Recognition of the full impact of COVID-19 is important to control the pandemic.
